# Refractory bleeding from a chest wall sarcoma: a rare indication for palliative resection

**DOI:** 10.1186/1749-8090-8-82

**Published:** 2013-04-12

**Authors:** Daniel J Weber, John J Coleman, Kenneth A Kesler

**Affiliations:** 1 Department of Surgery, Divisions of General Surgery, Indiana University School of Medicine, Indianapolis, IN 46202, USA; 2 Department of Surgery, Divisions of Plastic Surgery, Indiana University School of Medicine, Indianapolis, IN 46202, USA; 3 Department of Surgery, Divisions of Cardiothoracic Surgery, Indiana University School of Medicine, Indianapolis, IN 46202, USA

**Keywords:** Chest wall sarcoma, Refractory bleeding, Palliative resection

## Abstract

We report a case of a 57-year-old male who presented with an inoperable chest wall sarcoma due to numerous pulmonary metastases and was treated with chemotherapy and radiation therapy. The patient subsequently developed refractory bleeding from the chest wall tumor requiring palliative chest wall resection and reconstruction. The patient made an uneventful recovery however died from metastatic disease 8 months later. This case represents a very rare indication for palliative chest wall resection.

## Background

Soft tissue sarcomas represent less than 1% of malignancies occurring in the adult population and those arising from the chest wall comprise between 10% and 20% of these cases [1,2]. Management of chest wall sarcomas typically involves a multimodality approach including wide surgical resection with reconstruction [3]. In most cases, treatment is intended for curative purposes however occasionally palliative resection in the face of metastatic disease is needed. Local complications as a result of radiation therapy can occasionally occur such as skin ulceration, pain syndromes, and extensive tumor necrosis for which palliative surgery can improve the remaining quality of life [4]. We report a case of refractory bleeding from tumor necrosis after chemotherapy and radiation therapy that required operative intervention for palliation.

## Case presentation

**Figure 1:**
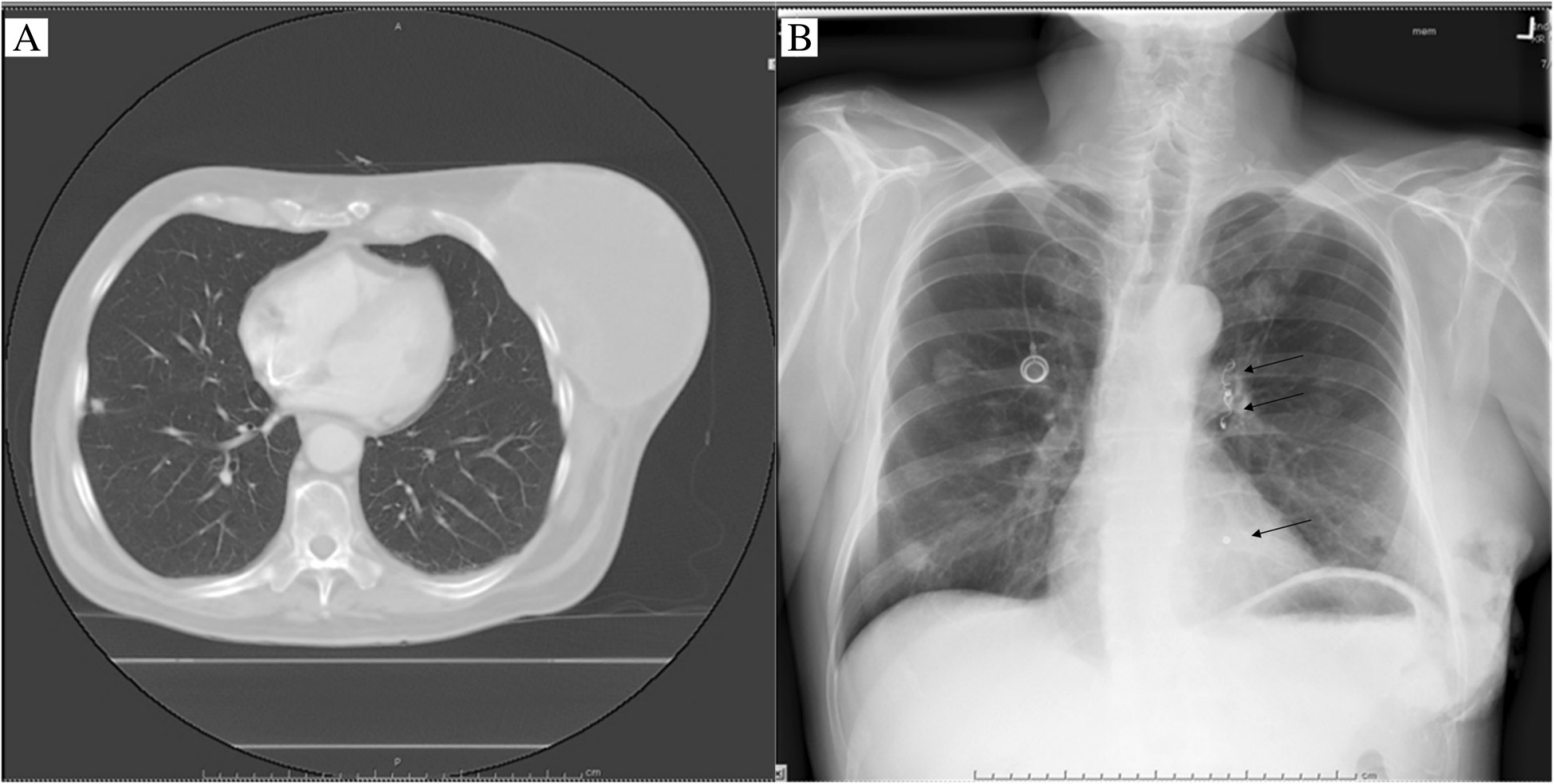
Pretreatment chest CT scan (A) showing left chest wall sarcoma and preoperative chest x-ray (B) with evidence of tumor and multiple embolization coils (dark arrows).

A 57-year-old Caucasian male with a 60-pack-year smoking history and no other co-morbidities or medical conditions initially presented to an outside hospital with a large left-sided chest wall neoplasm. (Figure 1A) Biopsy revealed a high-grade pleomorphic sarcoma. At the time of presentation, there were numerous pulmonary metastases and therefore he was not felt to be a surgical candidate. He received 3 cycles of doxorubicin and ifosfamide chemotherapy resulting in a modest radiographic response in the pulmonary metastases. There was no appreciable change in the primary neoplasm and he subsequently received radiation therapy to the chest wall with a total of 5,000 cGy over 25 treatments. Six weeks after receiving radiation, he developed significant skin and tumor ulceration resulting in diffuse bleeding refractory to multiple local hemostatic measures including cauterization and application of numerous topical agents. He required constant blood transfusions, a total of 8 units over a 12-week interval. Hematologic studies revealed no evidence of an underlying bleeding disorder. Attempts at percutaneous transarterial coil embolization of several chest wall arteries were subsequently performed however they were unsuccessful and the bleeding and skin ulceration worsened. (Figure 1B) As the patient’s metastatic disease was felt to be controlled, he was referred to our institution for urgent chest wall resection.

**Figure 2:**
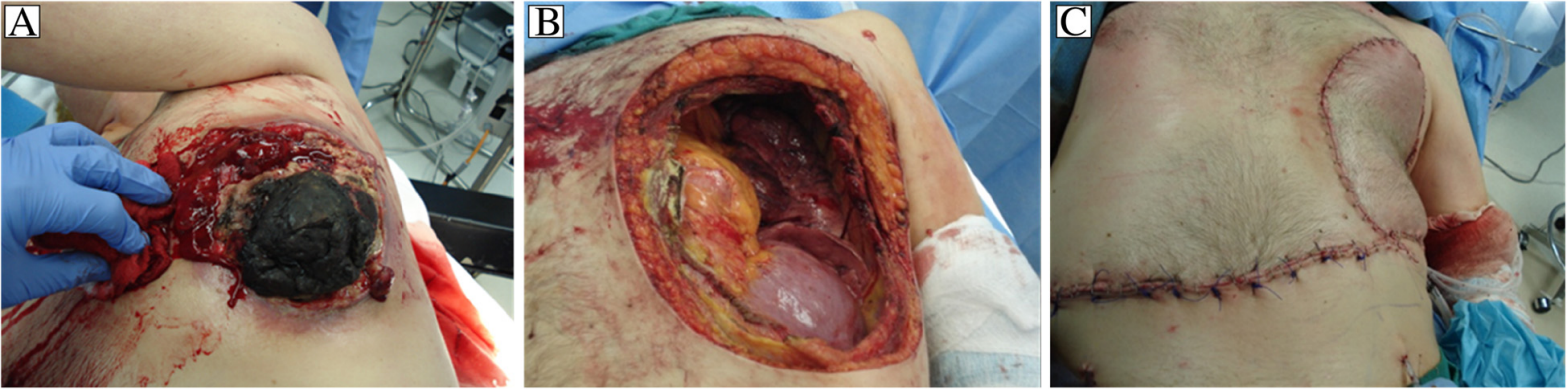
Bleeding necrotic chest wall sarcoma (A) with chest wall defect before (B) and after reconstruction (C).

At the time of surgery, wide chest wall excision was performed with an approximately 3 cm circumferential gross tumor free margin. Ultimately, ribs 4 through 8 were excised anteriorly from the costal cartilages to the anterior axillary line posteriorly. The resultant chest wall defect measured approximately 15 cm by 20 cm (Figure 2B). Chest wall reconstruction was accomplished with a double layer of prolene mesh covered by transverse rectus abdominus myocutaneous (TRAM) flap (Figure 2C). Pathologic analysis demonstrated an undifferentiated high-grade pleomorphic sarcoma with microscopic tumor free margins. The patient’s post-operative course was uncomplicated and he was discharged after eight days in improved condition. He did well for 6 months however developed metastatic disease to the bone and brain and died 8 months following surgery.

## Discussion

While surgical resection remains the standard of care for primary chest wall sarcomas, the role of a multimodality therapy approach appears promising. There is growing evidence to suggest chemotherapy may reduce systemic recurrence and improve survival [5]. Pulmonary metastatectomy can also be considered for curative purposes in select cases.

For inoperable patients with chest wall sarcomas, a combination of chemotherapy and local radiation is typically offered to control distant and local disease. Complications of radiation therapy may occur and include skin ulceration, pain syndromes, and primary tumor necrosis with or without superimposed infections [4]. While such complications are usually mild, on occasion, combined chest wall resection with reconstruction is indicated for palliation of severe complications with the goal of improving the remaining quality of life [6].

Significant and refractory bleeding from soft tissue sarcomas following radiation therapy appears however to be a rare event. A review of the literature (a PubMed search from 1990 to 2012 with index words: chest wall sarcoma, resection, bleeding) failed to identify any reports of chest wall resections performed for refractory bleeding. The decision to pursue palliative resection is certainly challenging for both patients and caregivers. When multiple efforts including arterial embolization failed to stop hemorrhage in our patient, chest wall resection and reconstruction clearly became necessary. Although the optimal timing of surgery is unknown in these situations, we speculate that earlier referral for resection could have been considered in this case.

## Conclusions

In summary, we encountered a rare case of significant and refractory bleeding from a chest wall sarcoma following radiation therapy. The high-grade and large size of the primary neoplasm may have contributed to this unusual complication. Bleeding refractory to conservative measures, along with other significant complications typically following radiation therapy, can be indications for palliative chest wall resection and reconstruction. Although seemingly radical in the face of incurable disease, surgery can allow an extended or improved quality of life.

## Consent

Telephone informed consent was obtained from the patient’s family for publication of this Case report and any accompanying images. A copy of the written consent is available for review by the Editor-in-Chief of this journal.

## Competing interests

The authors declare that they have no competing interest.

## Authors’ contributions

Each author contributed to the design and drafting of this manuscript. All authors read and approved the final manuscript.
